# Multiple Second to Fifth Carpometacarpal Fracture-Dislocations: A Case Report on the Surgical Management of a Rare Hand Injury

**DOI:** 10.7759/cureus.103378

**Published:** 2026-02-10

**Authors:** Sofía G Valdés-Medina, Ranulfo Romo-Rodríguez, Mario Villafán-Athié

**Affiliations:** 1 Orthopedics and Traumatology, American British Cowdray (ABC) Medical Center, Mexico City, MEX; 2 Orthopedics and Hand Surgery Unit, American British Cowdray (ABC) Medical Center, Mexico City, MEX; 3 Social Service, Faculty of Medicine, Universidad Panamericana, Mexico City, MEX

**Keywords:** bridge plate fixation, carpometacarpal joint dislocation, carpometacarpal joint fracture-dislocation, hand trauma management, high energy trauma

## Abstract

Multiple carpometacarpal (CMC) joint fracture dislocations involving the second to fifth rays are rare injuries, typically resulting from high-energy trauma. Due to the complex anatomy of the CMC joints and associated swelling, these lesions are frequently overlooked, leading to delayed diagnosis and suboptimal outcomes.

We report the case of a 21-year-old male patient who sustained simultaneous dislocations of the second through fifth carpometacarpal joints with associated fractures following a high-energy trauma to the hand due to a motorcycle accident. Initial radiographic evaluation confirmed dorsal displacement of the second to fifth metacarpal bases with fracture of the second and third metacarpal bases (Arbeitsgemeinschaft für Osteosynthesefragen (AO) 70D6(5b)), trapezoid avulsion fracture (AO 76.3.A), and hamate avulsion fracture (AO 74B). Close reduction under fluoroscopic guidance was attempted but proved unsuccessful due to the instability of the injury, requiring open reduction and internal fixation. A double dorsal surgical approach was used, and stabilization was achieved with Kirschner wires, a bridge plate, and an arthrodesis screw. Postoperative imaging demonstrated adequate joint alignment.

Multiple CMC fracture dislocations represent an uncommon injury pattern and require a high index of suspicion. Early diagnosis, appropriate imaging, and stable fixation are essential to restore joint congruency and prevent long-term complications such as chronic pain, instability, and post-traumatic early arthrosis. Given the inherent instability of these injuries, surgical management is frequently indicated.

Simultaneous fracture dislocations of the second to fifth CMC joints are rare and potentially disabling injuries. Prompt recognition and surgical stabilization can lead to satisfactory functional outcomes. This case highlights the importance of early intervention and appropriate fixation techniques in complex CMC joint injuries.

## Introduction

Carpometacarpal (CMC) joint fracture-dislocations are uncommon injuries, accounting for less than 1% of all hand traumas [1]. Dislocations involving multiple rays of the second to fifth CMCs are particularly rare and are typically associated with high-energy mechanisms, such as motor vehicle accidents or direct axial loading of the hand. The intrinsic stability provided by strong ligamentous structures and the complex anatomy of the CMC joints contribute to the infrequency of these injuries [1,2].

Diagnosis of multiple CMC dislocations remains challenging, as significant soft-tissue swelling and overlapping bony structures on standard radiographs may obscure the injury [1,2]. As a result, the lesions are frequently missed or diagnosed late, which may lead to poor functional outcomes, including chronic pain, joint instability, reduced grip strength, and post-traumatic osteoarthritis [3].

Early recognition and appropriate management are crucial to restoring joint congruency and hand function. While closed reduction may be attempted, these injuries are often inherently unstable, requiring surgical intervention with open reduction and internal fixation to achieve and maintain adequate alignment [3,4]. Associated carpal fractures, such as avulsion fractures of the trapezoid or hamate, further complicate management and reinforce the need for stable fixation [2].

The purpose of this case report is to highlight key surgical decision-making principles in the management of complex multiple CMC fracture-dislocations, including fixation strategy, the role of stabilizing the third CMC joint as the keystone of hand stability, and the importance of maintaining a high index of suspicion in polytrauma patients.

## Case presentation

A 21-year-old male patient presented to the emergency department after sustaining a high-energy motor vehicle accident. The patient was the rider of a motorcycle involved in a collision on a high-speed roadway. His medical history was significant for epilepsy, well-controlled with oxcarbazepine.

Upon arrival, the patient was managed according to the Advanced Trauma Life Support (ATLS) protocol. After initial stabilization and exclusion of life-threatening injuries, a focused evaluation of the hand was performed. Associated skeletal injuries included fractures of the zygomatic and maxillary bones; no other skeletal injuries were identified.

Examination of the hand revealed marked swelling, dorsal deformity, and pain-limited motion. Neurovascular status was intact. Given the high-energy mechanism, hand compartment syndrome was actively suspected and investigated through serial clinical examinations and was clinically excluded.

**Figure 1:**
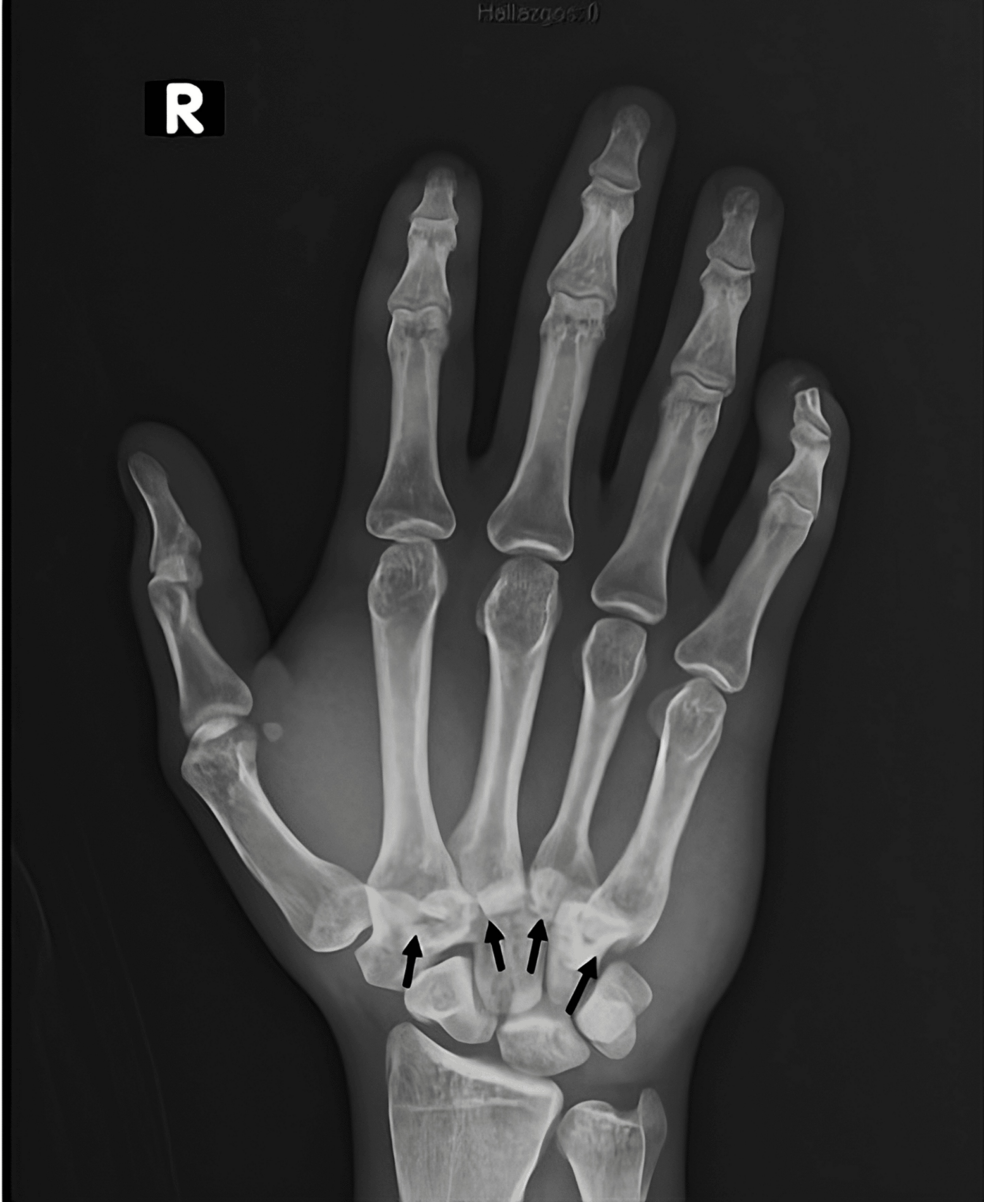
AP hand radiograph showing carpometacarpal fracture-dislocations of the second through fifth CMC joints AP: anteroposterior; CMC: carpometacarpal

**Figure 2:**
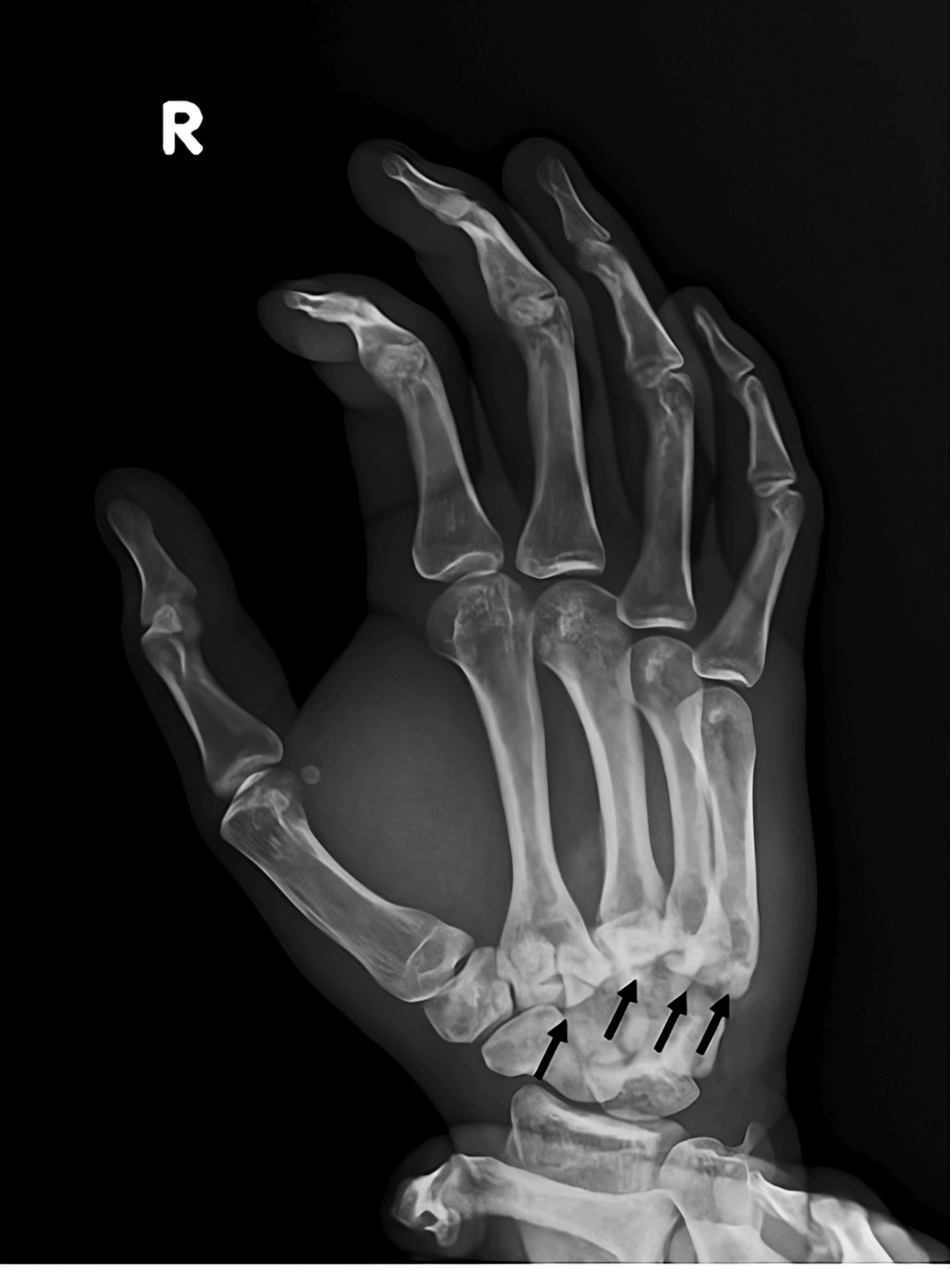
Oblique radiograph of the hand showing carpometacarpal fracture dislocations of the second through the fifth CMC joints CMC: carpometacarpal

Initial plain radiographs demonstrated simultaneous dorsal dislocations of the second through fifth carpometacarpal joints, with associated fracture of the base of the second and third metacarpals. Anteroposterior (Figure 1) and oblique (Figure 2) radiographs were obtained.

**Figure 3:**
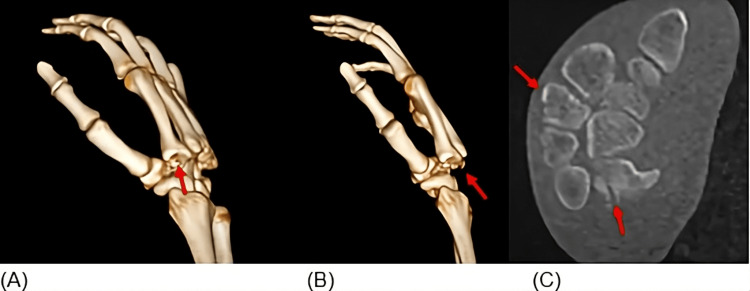
CT scan of the hand (A) and (B) show the 3D reconstruction, confirming the fracture dislocation with dorsal deviation. (C) In an axial cut of the CT, showing overlapping of the base of the second to fifth metacarpals with the distal row of the carpal bones and the avulsion fracture of the hamate.

A computed tomography (CT) scan of the hand with three-dimensional reconstruction was subsequently obtained, allowing better characterization of the injury. The CT scan confirmed the presence of avulsion fractures of the trapezoid (AO 76.3.A) and the hamate (AO74B), in addition to the multiple carpometacarpal dislocations (Figure 3).

Given the severity and inherent instability of the injury, closed reduction under fluoroscopic guidance was initially attempted; however, loss of reduction occurred, confirming persistent instability. Surgical management was therefore indicated and was performed five days after admission, following a period of close monitoring to rule out the development of compartment syndrome.

Open reduction was performed through double dorsal longitudinal approaches to the second-third and fourth-fifth carpometacarpal joints. Intraoperative findings confirmed joint incongruity and ligamentous disruption. The reduction was unstable, requiring Kirschner wire fixation of the fifth carpometacarpal joint, extending from the base of the fifth metacarpal to the hamate and capitate. A second parallel Kirschner wire was placed for additional stability. Fixation of the fourth metacarpal using two Kirschner wires from the base of the fifth metacarpal to the base of the fourth metacarpal was performed. Reduction of the third carpometacarpal joint was also performed.

**Figure 4:**
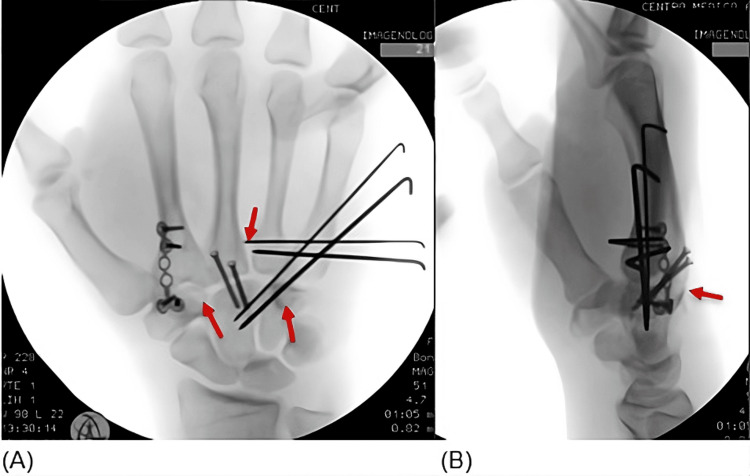
Postoperative AP (A) and lateral (B) radiographs showing carpometacarpal alignment AP: anteroposterior

Relative joint integrity was noted; therefore, fixation was performed using two screws from the base of the third metacarpal to the capitate, preserving the articular cartilage. Bridge fixation was applied. Screw fixation was preferred because the third carpometacarpal joint is the most stable carpometacarpal articulation and represents the keystone of hand stability; therefore, rigid fixation at this level provides a stable reference for overall hand alignment. Reduction of the second carpometacarpal joint was performed. An associated trapezoid avulsion fracture was identified and stabilized with plate fixation from the second metacarpal to the trapezoid, preserving the articular cartilage. Repair of the carpometacarpal ligaments and reduction of the hamate avulsion fracture were performed to maintain alignment and restore carpal stability (Figure 4).

**Figure 5:**
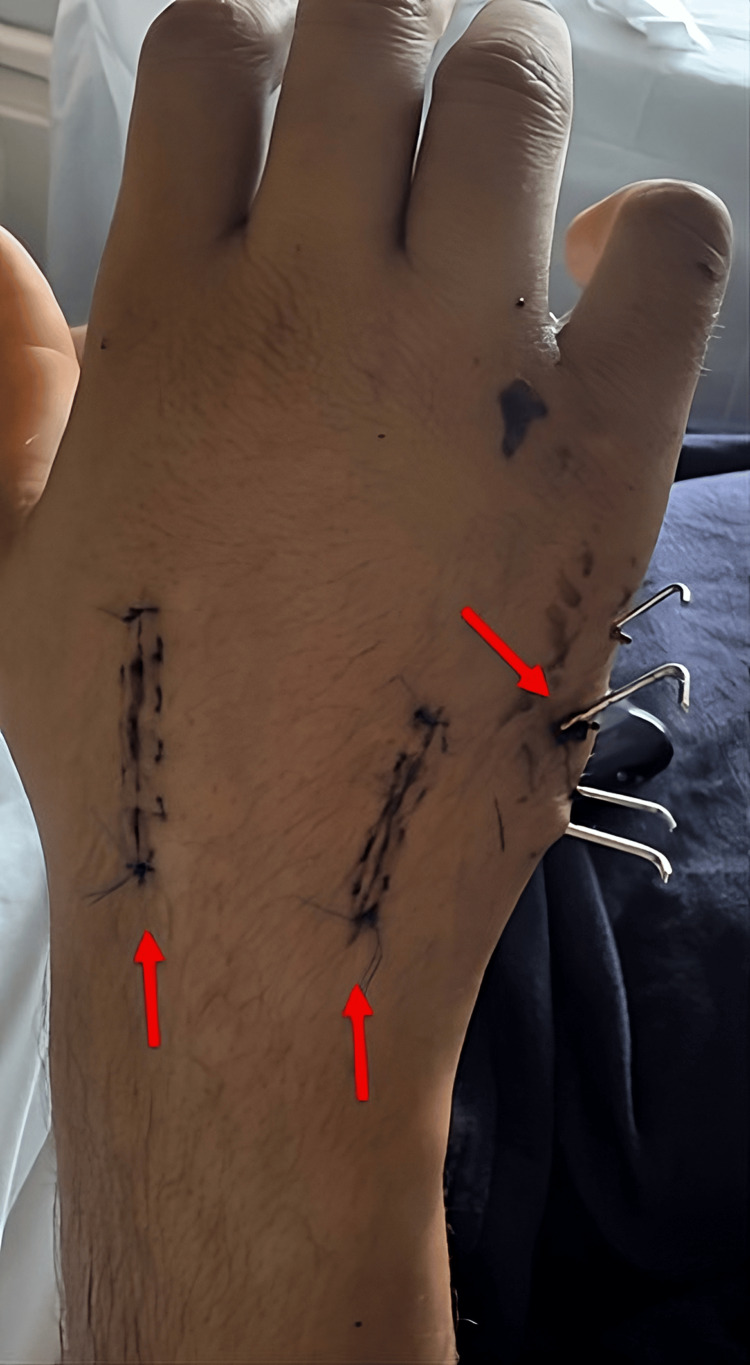
One-week postoperative follow-up clinical image, showing the double dorsal approach and the percutaneous Kirschner wires

Postoperative radiographs demonstrated satisfactory reduction and stable fixation of all involved carpometacarpal joints. The patient was immobilized postoperatively with a forearm-based palmar splint in combination with a Bunnell-type dressing and followed with serial clinical and radiographic evaluations. At six weeks postoperatively, the Kirschner wires were removed without complications. At follow-up, maintenance of reduction and satisfactory clinical progression were observed (Figure 5).

## Discussion

Multiple CMC joint fracture-dislocations involving the second through fifth rays are rare injuries, typically resulting from high-energy trauma [1]. The strong ligamentous architecture and interlocking anatomy of the CMC joints provide inherent stability, making simultaneous dislocations uncommon [3]. When they do occur, these injuries are often associated with carpal fractures, reflecting the magnitude of the traumatic force involved.

Early diagnosis of multiple CMC dislocations remains challenging. Significant soft tissue swelling and overlapping bony structures on standard radiographs may obscure the injury, leading to missed or delayed diagnosis [2-4]. In the present case, computed tomography with three-dimensional reconstruction was essential to accurately identify the associated avulsion fractures of the trapezoid and hamate and to fully characterize the extent of the injury. Advanced imaging should therefore be considered when clinical suspicion persists despite inconclusive plain radiographs.

Consistent with previous reports, this injury pattern proved inherently unstable, necessitating surgical stabilization after failed closed reduction [5,6]. Compared with previously published cases, our approach emphasizes rigid fixation of the third CMC joint to restore longitudinal stability while preserving articular cartilage.

Management of multiple CMC dislocations is controversial; however, most authors agree that these injuries are inherently unstable and frequently require surgical stabilization [5]. Although closed reduction may be attempted initially, loss of reduction is common, as observed in this case. Open reduction allows direct visualization of joint congruity, assessment of ligamentous integrity, and stable fixation, particularly in the presence of associated carpal fractures [2].

Various fixation techniques have been described, including Kirschner wires, dorsal plates, and screw fixation [5,6]. In this case, a combination of Kirschner wires, bridge plating, and screw fixation was used to achieve stable reduction while preserving the articular cartilage. This strategy provided sufficient stability to allow early maintenance of reduction, with subsequent removal of the Kirschner wires at six weeks postoperatively. Repair of the carpometacarpal ligaments further contributed to joint stability.

Early operative intervention, exclusion of compartment syndrome, and tailored fixation strategies are critical to optimizing outcomes and minimizing long-term complications. Delayed or inadequate treatment of multiple CMC dislocations can result in poor functional outcomes, including chronic pain, grip strength reduction, joint stiffness, instability, and post-traumatic osteoarthritis [1-6]. Prompt recognition, appropriate imaging, and early surgical stabilization are therefore critical to optimize outcomes [5]. The satisfactory postoperative course observed in this patient supports the use of early operative management in complex CMC joint injuries.

This case adds to the limited literature on simultaneous second through fifth CMC dislocations associated with carpal avulsion fractures and highlights the importance of a systematic trauma evaluation, advanced imaging, and tailored surgical fixation to restore hand anatomy and function.

## Conclusions

Simultaneous fracture-dislocations of the second through fifth carpometacarpal joints are rare injuries typically associated with high-energy trauma and frequently accompanied by carpal fractures. Due to their inherent instability and the risk of missed diagnosis, a high index of suspicion and appropriate imaging, including computed tomography, are essential for accurate assessment.

Operative treatment should not be considered mandatory in all cases. However, given the inherent instability of these injuries, surgical management is frequently indicated, particularly in cases with multiple carpometacarpal dislocations or associated fractures. Early surgical management with open reduction, stable fixation, and ligament repair can restore joint alignment and provide satisfactory clinical outcomes. This case underscores the importance of prompt recognition and individualized fixation strategies in the treatment of complex carpometacarpal joint injuries.
